# FABP5 enhances malignancies of lower‐grade gliomas via canonical activation of NF‐κB signaling

**DOI:** 10.1111/jcmm.16536

**Published:** 2021-04-09

**Authors:** Yichang Wang, Alafate Wahafu, Wei Wu, Jianyang Xiang, Longwei Huo, Xudong Ma, Ning Wang, Hao Liu, Xiaobin Bai, Dongze Xu, Wanfu Xie, Maode Wang, Jia Wang

**Affiliations:** ^1^ Department of Neurosurgery The First Affiliated Hospital of Xi'an Jiaotong University Xi'an China; ^2^ Center of Brain Science The First Affiliated Hospital of Xi'an Jiaotong University Xi'an China; ^3^ Department of Neurosurgery The First Hospital of Yulin Yulin China

**Keywords:** FABP5, lower‐grade glioma, malignancy, NF‐κB, tumour recurrence

## Abstract

Low‐grade gliomas (LGGs) are grade III gliomas based on the WHO classification with significant genetic heterogeneity and clinical properties. Traditional histological classification of gliomas has been challenged by the improvement of molecular stratification; however, the reproducibility and diagnostic accuracy of LGGs classification still remain poor. Herein, we identified fatty acid binding protein 5 (FABP5) as one of the most enriched genes in malignant LGGs and elevated FABP5 revealed severe outcomes in LGGs. Functionally, lentiviral suppression of FABP5 reduced malignant characters including proliferation, cloning formation, immigration, invasion and TMZ resistance, contrarily, the malignancies of LGGs were enhanced by exogenous overexpression of FABP5. Mechanistically, epithelial‐mesenchymal transition (EMT) was correlated to FABP5 expression in LGGs and tumour necrosis factor α (TNFα)‐dependent NF‐κB signalling was involved in this process. Furthermore, FABP5 induced phosphorylation of inhibitor of nuclear factor kappa‐B kinase α (IKKα) thus activated nuclear factor kappa‐B (NF‐κB) signalling. Taken together, our study indicated that FABP5 enhances malignancies of LGGs through canonical activation of NF‐κB signalling, which could be used as individualized prognostic biomarker and potential therapeutic target of LGGs.

## 1 INTRODUCTION

Gliomas are infiltrative neoplasms arise in central nervous system of adults exhibiting remarkable genetic heterogeneity, alternative biological signatures and clinical properties.^1^ Gliomas are histologically classified into four grades based on the morphology and immunohistochemistry signatures according to the World Health Organization (WHO) classification. Moreover, WHO grades II and III gliomas, including astrocytomas, oligodendrogliomas and mixed oligoastrocytomas, are distinguished from grade IV (glioblastoma, GBM) and defined as lower‐grade gliomas (LGGs), consistent with The Cancer Genome Atlas (TCGA) categorization.^2^ Accumulating evidence has shown that the progression and clinical behaviours of LGGs varies from each other due to their variable genetic characters, which resulted in completely diversity of survival. Some LGGs remain progression‐free for years; however, other subset of LGGs are highly invasive and progress to GBMs within months thus resulted in rapid recurrence and treatment failure within months.^3^ A wide range of efforts including genetic identification and image‐based classification has been made during the past decade to precisely evaluate the malignancy and reveal the prognosis of LGGs; however, the reproducibility and diagnostic accuracy of LGGs classification still remain extremely poor.^4^, ^5^ Therefore, developing reliable and sensitive prognostic biomarkers becomes essential for improving the individualized clinical management among LGG patients.

Recent studies based on genome‐sequencing and epigenetics analysis demonstrate molecular alterations and chromosomal mutations are associated with clinical outcomes and provide superior prognostication in LGGs and have led to substantial changes in the updated WHO categorization.^6^, ^7^ Evidence strongly suggests that mutations in isocitrate dehydrogenase (IDH) 1 and IDH2 could be found in more than 70% of LGGs and predict a more favourable prognosis, contrarily, patients with IDH wild‐type LGGs show significantly dismal outcomes.^6^ Moreover, Reuss et al^8^ demonstrate that the majority of IDH wild‐type LGGs show extremely severe survival and should be identified as unrecognized GBMs, indicating that IDH mutation might be responsible for malignant subtype transition in LGGs. Following the detection of IDH mutations in LGGs, combined deletion of chromosome arms 1p and 19q (1p/19q co‐deletion), which are frequently observed in oligodendrogliomas, are found to be correlated with prolonged survival in LGGs.^9^ Researchers find that LGGs with IDH mutation and 1p/19q co‐deletion are more sensitive to radio treatment and chemotherapy and reveal more favourable survival when compared to LGGs without these alterations.^10^ Comparatively, patients with IDH wild‐type and 1p/19q intact LGGs present a significantly decreased median survival time of 1.7 years, which is similar to GBM patients.^11^

Although IDH and 1p/19q status has been applied as major classifiers for LGGs, the prognostic evaluation for those genetic alterations is still ambiguous. Chan et al^12^, ^13^ reported that IDH wild‐type LGGs do not have a universally poor survival since the existence of heterogeneity among LGGs. LGGs with EGFR amplification, TERT promoter mutation or H3F3A mutation exhibit more severe prognosis despite the genetic background of wild‐type IDH.^14^ Additionally, a long‐term randomized clinical trial also indicates that benefit of procarbazine, lomustine, vincristine (PCV) chemotherapy on LGGs is not limited to the 1p/19q co‐deleted cases while the survival of patients with 1p/19q non co‐deletion is markedly prolonged by PCV treatment.^15^ Moreover, IDH mutation or 1p/19q alterations could only be used as prognostic indicators for LGGs but either of them could be used for potential molecule‐targeted drug designing. Further studies should be performed to clarify the underlying mechanism and develop novel therapeutic targets for LGGs.

In this study, we identified fatty acid binding protein 5 (FABP5) was significantly enriched in malignant LGGs which were defined by IDH^WT^ and 1p/19q^Non co‐del^. Additionally, elevated FABP5 could be observed in higher WHO grade LGGs and revealed severe outcomes. Functionally, lentiviral suppression of FABP5 reduced malignant characters including proliferation, cloning formation, immigration and invasion, contrarily, the malignancies of LGGs particularly chemotherapy resistance to temozolomide (TMZ) was markedly enhanced by exogenous overexpression of FABP5. With bioinformatics analysis, epithelial‐mesenchymal transition (EMT) was identified as one of the most correlated cellular process to FABP5 expression in LGGs. Gene set enrichment analysis (GSEA) demonstrate that increased FABP5 was closely related to tumour necrosis factor α (TNFα)‐dependent NF‐κB signalling. Results from mechanism study confirmed that FABP5 promoted malignant signatures of LGGs via inducing phosphorylation of inhibitor of nuclear factor kappa‐B kinase α (IKKα) thus activated nuclear factor kappa‐B (NF‐κB) signalling. Taken together, our study indicated that FABP5 enhances malignancies of LGGs through canonical activation of NF‐κB signalling, which could be used as individualized prognostic biomarker and potential therapeutic target of LGGs.

## 2 MATERIALS AND METHODS

### 2.1 Ethics

All the usage of patient samples in this study is approved by the Scientific Ethics Committee of the First Affiliated Hospital of Xi'an Jiaotong University, Xi'an, China (approve no. 2016‐18). All samples have obtained necessary consent and were embedded in paraffin blocks as previously described. Totally, 63 glioma samples and three non‐tumour tissue samples (from tumour edge) were collected from patients underwent surgical operations from 2016 to 2020. The basic information about 63 glioma patients was described in Table S1. And the WHO grade information, IDH and 1p19q status for patient samples, which were performed for a series of experiments in *vivo* and in *vitro*, were described in Table S2.

### 2.2 Differential gene expression analysis

Gene expression datasets and relevant clinical data of LGG were respectively extracted from Chinese Glioma Genome Atlas (CGGA, mRNASeq_693, http://cgga.org.cn/) and GDC Data Portal (https://portal.gdc.cancer.gov/). These gene expression profile data were pre‐processed by background correction, gene symbol transformation and normalization using R programming (vision 4.0.0). The limma package was utilized for screening the differentially expressed genes (DEGs) in these datasets. The expression difference of individual gene was identified by Log_2_ (Fold change) and adjusted *P* value, in which Log_2_ FC > 2 with an adjusted *P* value < .05 was identified as a DEG.

### 2.3 Time‐dependent receiver operating characteristic (ROC) curve

Time‐dependent ROC analysis was performed according to previous reference for further measurement of the predictive performance among the candidate genes.^16^ Briefly, survival data and gene expression data were extracted from CGGA_mRNASeq_693 dataset. False positive rate and true positive rate were used as *x* axis and *y* axis, respectively, thus the performance of a gene candidate was evaluated by the area under the ROC curve (AUC) in which a higher AUC value indicates a better performance.

### 2.4 Least absolute shrinkage and selection operator (LASSO) regression

To further narrow the scope of the candidate genes, we adopted the LASSO binary logistic regression model and multivariate Cox regression according to previous reference.^17^ The LASSO logistic regression was applied to genome‐wide association study (GWAS) to identify genomic locus associated with complex disease, and it can be directly employed in quantitative trait locus mapping for binary traits as shown in our simulation study and real data analysis. A coefficient profile figure was produced against the ln (λ) sequence. Cross‐validation (CV) for tuning parameter (λ) selection was analysed via one standard error criteria. Two dotted vertical lines were presented at the optimal values by using minimum criteria and the one standard error of the minimum criteria (1‐*SE* criteria). Genes with ln (λ) > 1.725 were selected, which is 1‐*SE* criteria. Then the LASSO coefficient profile of nine candidate genes was calculated with CGGA cohort.

### 2.5 Quantitative RT‐PCR (qRT‐PCR)

qRT‐PCR assays were conducted as previously described.^18^ In brief, total RNA was extracted using RNeasy mini kits according to the manufacturer's instruction, and the concentration of RNA was determined by Nanodrop 2000. qRT‐PCR was performed following the synthesis of cDNA according to the standard protocols. GAPDH was used as an internal control. Relative mRNA expressions were calculated by 2^−ΔΔt^ method. The primer sequences were described in Table. S3.

### 2.6 Western blot

Western blot analysis was performed as previously described^18^ and each western blot was repeated at least three times. Antibodies used in this study were shown as below: anti‐FABP5 primary antibody was purchased from Abcam (ab84028). Anti‐β‐actin primary antibody was purchased from Abcam and served as an internal control (ab115777). Anti‐IKKα primary antibody was purchased from Cell Signaling Technology (#11930). Anti‐p‐IKKα (Ser176) primary antibody was purchased from Biorbyt (orb544342). Anti‐IKβ (ab32518) and anti‐NF‐κB (ab32360) primary antibodies were purchased from Abcam. Anti‐Rabbit IgG (#7074) and Anti‐Mouse‐IgG (#7076) were purchased from Cell Signaling Technology and served as negative controls. The raw unedited bands of all western blot images were showed in the Figure S7.

### 2.7 Immunohistochemistry (IHC)

Immunohistochemistry was performed as previously described.^19^ Briefly, anti‐FABP5 primary antibodies were purchased from Thermo Fisher Scientific (PA5‐76920). Goat anti‐rabbit IgG was purchased from Abcam (ab97051) and served as secondary antibody. Tissues embedded with paraffin were cut into 4 mm sections followed by deparaffinized, rehydrated and stained with primary antibodies at 4℃ overnight. The slides were then incubated with secondary antibodies and stained with DAB. All glioma samples used in this study had been pathologically diagnosed and the recurrence was confirmed by computed tomography (CT) or magnetic resonance imaging (MRI). Nuclei were counterstained with haematoxylin.

### 2.8 German Immunohistochemistry Score (GIS)

GIS was used to measure the expression of FABP5 as previously described.^19^ Immunoreactivity score = positive cell score × staining intensity score. The positive cell score was calculated as below: 0, negative; 1, <10% positive; 2, 11%‐50%; 3, 51%‐80%; 4, >80%. Staining intensity score was graded as below: 0, negative; 1, weakly positive; 2, moderately positive; 3, strongly positive. Immunoreactivity score > 5 was considered as high expression and ≤ 5 were defined as high and low expression, respectively.

### 2.9 Cell cultures

LGG‐derived primary culture cells 0708, 7419 and 7624 were obtained from LGGs patient samples that underwent surgical resection in the Department of Neurosurgery, the First Affiliated Hospital of Xi'an Jiaotong University. Fresh surgical LGG specimens were rinsed in PBS in order to remove adhering blood and visible necrotic portions. Mechanical and enzymatic tissue dissociation by trypsin solution was carried out in order to obtain single cell suspensions. Single cells were seeded into DMEM‐F12 containing 15% FBS, 2% B27 and antibiotics (100 U/ml penicillin and 100 mg/ml streptomycin) and were incubated in sterile conditions at 37°C in a 5% CO_2_ humidified atmosphere and the complete medium was totally replaced every 2‐3 days. Only the early passages of the LGG primary cultures were used as in vitro cell model and cell viability was determined using the trypan blue methods. NHA cell line was gifted from Dr Jianxin Liu and cultured in DMEM‐F12 containing 10% FBS with antibiotics (100 U/ml penicillin and 100 mg/ml streptomycin). Cell Counting Kit‐8 (CCK‐8) methods were used to evaluate cell proliferation according to the manufacturer's protocol.

### 2.10 *In* *vitro* cell viability assays

Cell viability assays were conducted as previously described.^19^ Briefly, cell number was calculated by cell counter with trypan blue, and cells were seeded into 96‐cell plates after suspended adequately at a density of 2 × 10^3^ cells/100uL per well and cultured for 24 hours with 5% CO_2_ at 37℃. These cell lines received indicated in vitro chemotherapy using corresponding concentration of TMZ. At last, cell number was counted by Cell Counting Kit‐8 (CCK‐8) and IC50 was calculated by SPSS 22.0.

### 2.11 Lentivirus production and transduction

Lentivirus production and transduction were performed as described previously.^18^, ^19^ The lentiviral overexpression/knock‐down of FABP5 was designed and synthesized by Genechem (Shanghai, China). These lentiviruses were introduced into LGG cells according to manufacturer's instruction. Stable clones transduced with FABP5 and shFABP5 were selected for 4 weeks by puromycin. Lentiviral particles packaging the shRNA are targeting FABP5 (shFABP5#1: CCGGATTGAAAGATGGGAAATTAGTCTCGAGACTAATTTCCCATCTTTCAATTTTTTTG and shFABP5#2: CCGGGATGGGAAGGAAAGCACAATACTCGAGTATTGTGCTTTCCTTCCCATCTTTTTG).

### 2.12 Colony formation assay

Colony formation assays were conducted to detect the tumorigenic potential of LGG cells. 0708 or 7419 cells transduced with FABP5 overexpression lentivirus, shFABP5 lentivirus or empty vector were seeded into 6‐well plates under indicating conditions. After cultured for 14 days to form colonies, these cells were fixed with methanol and stained with methylene blue. Number of clones was counted to evaluate the colony forming ability.

### 2.13 Wound healing assay

The wound healing assay is used to study the migratory ability of 0708 and 7419 cells. Cells were seeded in 6‐well plates after adequately suspended. Afterwards, a sterile pipette tip was used to produce a wound line when cells reached the appropriate confluence. Images were taken under inverted microscope at the same location at 7 days after seeding. The leading edges were marked by white lines, and the relative distance of the borders was measured by Image J software.

### 2.14 Matrigel invasion assays

The matrigel invasion assays were performed to assess the invasion ability of 0708 or 7419 cells, 1 × 10^5^ cells pre‐transduced with indicating lentivirus were suspended sufficiently and seeded into the upper chamber of ThinCert cell culture inserts (8 μm pore size, Greiner Bio‐One). For cell invasion assays, the inserts were coated with 100 µl of 1 mg/ml growth factor depleted matrigel (Corning) before adding the transfected colon cancer cells. 500 µL serum‐free medium was added to the lower chamber and replaced 24 hours later by DMEM‐F12 containing 10% FBS. After incubation for 96 hours for invasion, inserts were removed, cells were fixed in 4% paraformaldehyde and methanol, and stained using 0.1% crystal violet. The non‐migrated cells were slightly removed by cotton swabs from the upper surface of the filters, and photomicrographs of invaded cells were taken.

Gene ontology (GO) analysis, Gene set enrichment analysis (GSEA) and Gene set variation analysis (GSVA).

The gene expression profiles of previously mentioned datasets were read into R programming and pre‐processed with background correction, gene ID transformation and normalization. Furthermore, these data were ordered by the expression level of FABP5 to divide all the LGG samples into two groups (FABP5^High^ and FABP5^Low^) by quartile cutoff. Subsequently, the limma package was used to perform DEGs analysis. Afterwards, the screened DEGs by log_2_FC > 2 and adjusted *P* value < .05 were used to perform gene ontology (GO) annotation using the Database for Annotation, Visualization and Integrated Discovery online tool (DAVID, https://david.ncifcrf.gov/), and GSVA package and GSEA software were used to validate, and visualized results using clusterprofiler package.

Besides, the expression profiles of LGG were uploaded into the GSEA software strictly following the guideline of the software to elucidate the enriched KEGG pathways that were significantly enriched in FABP5 high groups with the number of permutations set at 1000.

### 2.15 Statistical analysis

All the results in this study were presented as mean ± SD (standard deviation). The number of independent replications is presented in the figure legends. Two‐tailed *t* tests were conducted to evaluate statistical differences and one‐way ANOVA following Dunnett's post‐test was used to compare the statistics among multiple groups. Kaplan‐Meier survival analyses were performed using log‐rank test. All statistical analysis was performed by GraphPad Prism 7 or SPSS 22.0 software, and statistical significance was defined as a two‐sided *P* value less than 0.05.

## 3 RESULTS

### 3.1 FABP5 is one of the most differentially expressed genes in malignant LGGs

**FIGURE 1:**
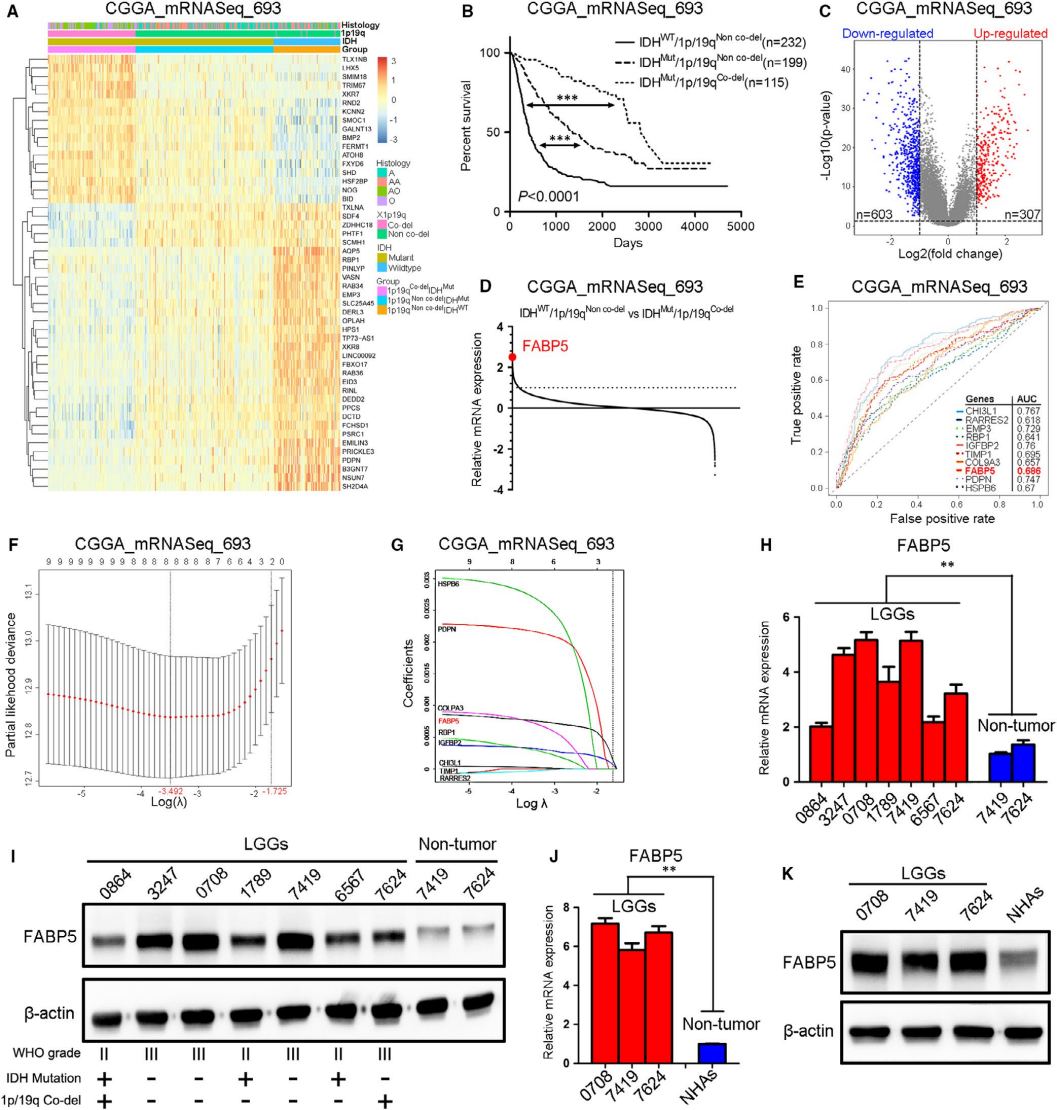
FABP5 is one of the most differentially expressed genes in malignant LGGs. A, Hierarchical bi‐clustering analysis with CGGA dataset indicated that significant gene signatures in LGGs classified by IDH and 1p/19q status. B, Kaplan‐Meier survival analysis for LGGs classified by IDH and 1p/19q status using CGGA dataset (*P* < .0001, with log‐rank test). C, Differentially expressed gene analysis in IDH^WT^/1p/19q^Non co‐del^ LGGs versus IDH^Mut^/1p/19q^Co‐del^ LGGs using CGGA database. D, Dot plot showed differentially expressed genes in IDH^WT^/1p/19q^Non co‐del^ LGGs versus IDH^Mut^/1p/19q^Co‐del^ LGGs using CGGA database. E, ROC analysis for differentially expressed gene candidates in CGGA LGGs database. F‐G, LASSO logistic regression for 8 most differentially expressed gene candidates in LGGs CGGA database. H‐I, qRT‐PCR H, and western blotting analysis I, of FABP5 expression in LGG tumour tissues and non‐tumour tissues. β‐actin served as an internal control. (**P < .01, n = 3, with one‐way ANOVA followed by Dunnett's post‐test). J‐K, qRT‐PCR J, and western blotting analysis K, of FABP5 expression in LGGs primary cultured cells and NHAs cells. β‐actin was used as an internal control. (**P < .01, n = 3, with one‐way ANOVA followed by Dunnett's post‐test)

To further identify the genetic heterogeneity and malignant signatures among LGGs, IDH and 1p/19q status was used as specific classifier of LGGs. After removal of absent expression and clinical data, totally 546 LGGs samples extracted from the CGGA_mRNASeq_693 dataset were divided into three subgroups which exhibited significantly genetic diversity (Figure 1A). Additionally, Kaplan‐Meier analysis was performed among the three subgroups and the results indicated that LGGs patients with IDH wild‐type and 1p/19q non co‐deletion had the most severe overall survivals while patients with IDH mutation and 1p/19q co‐deletion exhibited prolonged survivals, which was consistent with the WHO classification (Figure 1B). Furthermore, differential gene expression analysis was performed between IDH^WT^/1p19q^Non co‐del^ and IDH^Mut^/1p19q^Co‐del^ samples to explore the most significant prognosis‐related genes (Figure 1C). The results indicated that FABP5 was one of the most remarkably elevated genes in IDH^WT^/1p19q^Non co‐del^ samples (Figure 1D). Moreover, the prognostic capacity of the all genes was further demonstrated by the AUC of the time‐dependent ROC curve. As shown in Figure 1E, FABP5 was one of the most appropriate prognostic genes compared with other candidates. To further narrow the scope of the candidate genes, LASSO logistic regression was performed.Nine selected candidate DEGs featured coefficients (not zero) in a further LASSO logistic regression model in which the selected genes were required to appear 1000 times of 1000 repetitions. Finally, two genes (FABP5 and IGFBP2) were selected as prognostic gene candidates for LGGs (Figure 1F,G).

To further investigate the expression of FABP5 in LGGs, qRT‐PCR analysis was performed by using LGG samples (0864, 3247, 0708, 1789, 7419, 6567 and 7624) compared with non‐tumour samples deriving from the matched para‐cancerous tissues (7419 and 7624). The results demonstrated that FABP5 was significantly enriched in LGGs compared to non‐tumour tissues (Figure 1H), which is consistent with the results from western blot analysis (Figure 1I and Figure S1A). Additionally, both qRT‐PCR (Figure 1J) and western blot analysis (Figure 1K and Figure S1B) demonstrated that FABP5 was highly expressed in early passages of patient tumour‐derived LGGs primary cultures compared to normal human astrocytes. Taken together, these data suggested that FABP5 was one of the most differentially expressed genes in malignant LGGs.

### 3.2 Elevated FABP5 expression revealed poor prognosis in LGGs

**FIGURE 2:**
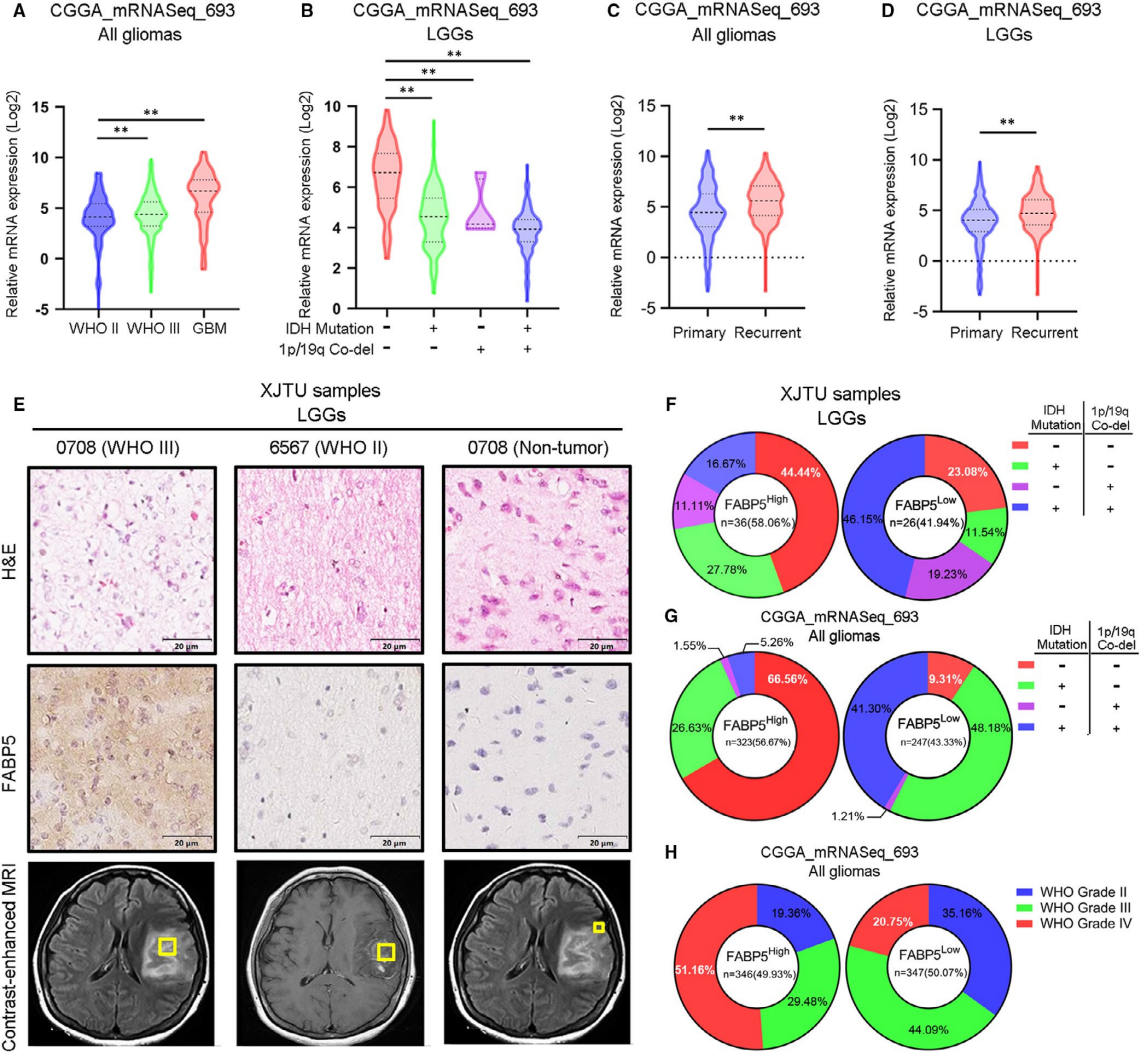
FABP5 expression was elevated in malignant LGGs. A, Expression of FABP5 in gliomas according to WHO classification by using CGGA database (**P < .01, with one‐way ANOVA followed by Dunnett's post‐test). B, Expression of FABP5 in LGGs based on IDH and 1p/19q status by using CGGA database (**P < .01, with one‐way ANOVA followed by Dunnett's post‐test). C‐D, Expression of FABP5 in primary or recurrent gliomas C, and LGGs D, by using CGGA database (**P < .01, with one‐way ANOVA followed by Dunnett's post‐test). E, Representative IHC images of FABP5 expression in glioma samples and non‐tumour tissues (Upper panel: H&E staining, Middle panel: FABP5 expression, Lower panel: MRI scanning). F‐H, Proportion distribution for FABP5 expression in glioma samples. Samples were clustered by IDH and 1p/19q status (F: patient samples, G: CGGA database) and WHO grade (H: CGGA database)

To gain more insight into clinical relevance of FABP5 expression in LGGs, CGGA and TCGA glioma databases were utilized to investigate the expression pattern of FABP5 in different pathological subtypes. The result indicated that FABP5 was increased in LGGs with higher WHO grade, particularly in GBM, compared to those with lower WHO grade (Figure 2A). Additionally, FABP5 was highly expressed in IDH^WT^ and 1p/19q^Non co‐del^ LGGs, which was considered as the most lethal subtype of LGGs (Figure 2B). We then explored the correlation between FABP5 expression and tumour recurrence. FABP5 was overexpressed in recurrent gliomas (Figure 2C), consistently, was significantly enriched in recurrent LGGs (Figure 2D). IHC staining was performed by using samples derived from patients underwent surgical resection in our institution. The results indicated that FABP5 was predominantly enriched in the cytoplasm of glioma cells, while no significant staining was observed in the non‐tumour tissues (Figure 2E). In addition, LGGs were divided into two groups according to the GIS of FABP5 expression. The data indicated that FABP5 were likely to be enriched in IDH^WT^ and 1p/19q^Non co‐del^ LGGs compared to other two subgroups (IDH^WT^ and 1p/19q^Non co‐del^ samples accounted for 44.44% in FABP5^High^ samples and accounted for 23.08% in FABP5^Low^ samples) (Figure 2F). Consistent results could be addressed when analysing CGGA dataset (Figure 2G). Moreover, increased FABP5 expression could be observed along with the malignancy elevation of gliomas in CGGA dataset (Figure 2H). Altogether, FABP5 elevation was closely correlated with higher histological grade and malignant subtypes of LGGs.

**FIGURE 3:**
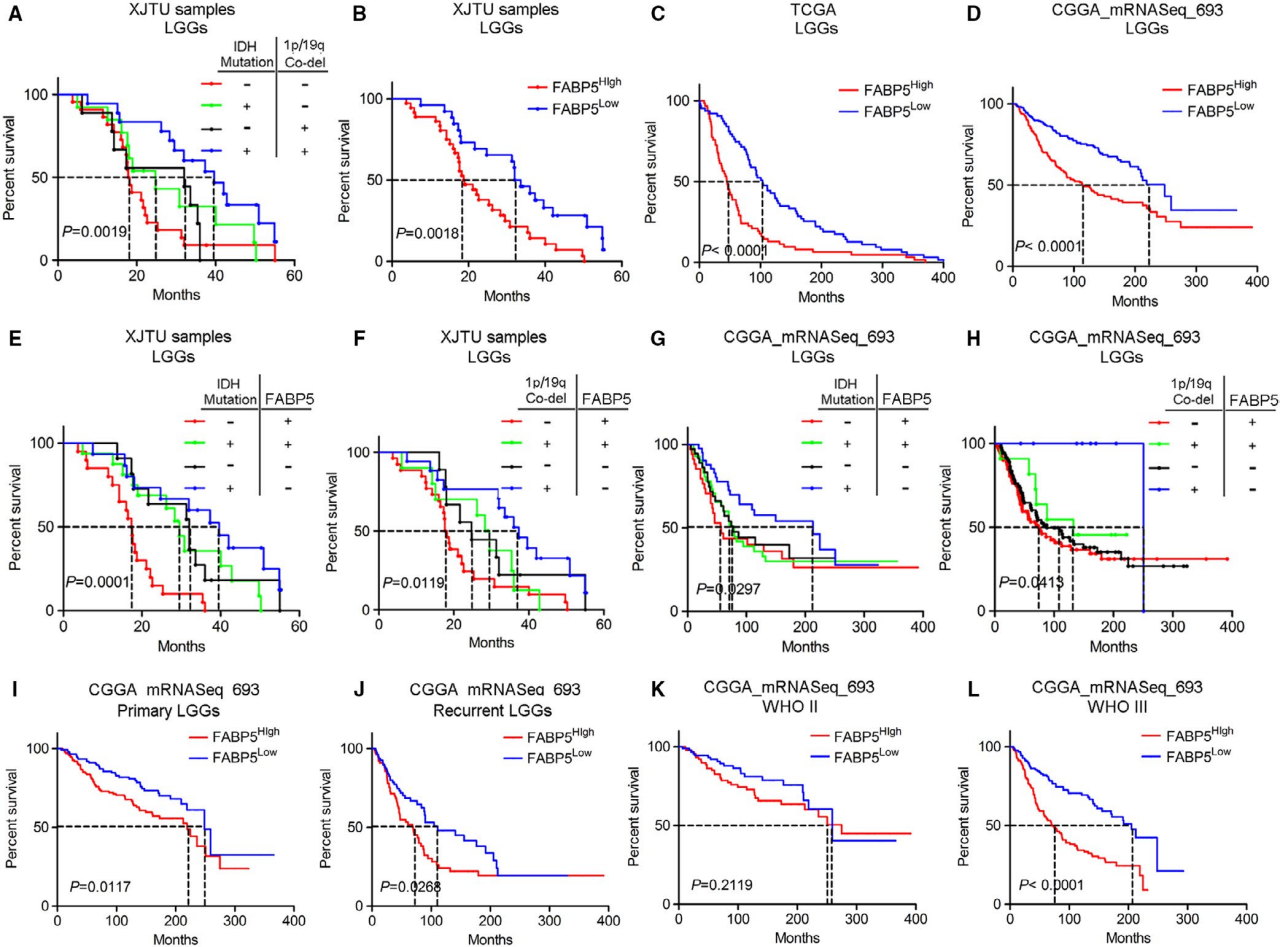
Enriched FABP5 revealed severe prognosis in LGGs. A, Kaplan‐Meier survival analysis for LGGs classified by IDH and 1p/19q status (*P* = .0019, with log‐rank test). B‐D, Kaplan‐Meier survival analysis for FABP5 expression in LGGs by using patient samples from our institution, TCGA and CGGA databases (B: *P* = .0018, C‐D: *P* < .0001, with log‐rank test). E‐H, Kaplan‐Meier survival analysis for FABP5 expression combined with IDH or 1p/19q status in LGGs by using patient samples from our institution and CGGA database (E: FABP5 combined with IDH mutation in LGG samples from our institution, *P* = .0001; F: FABP5 combined with 1p/19q co‐deletion in LGG samples from our institution, *P* = .0119; G: FABP5 combined with IDH mutation in LGG samples from CGGA database, *P* = .0297; H: FABP5 combined with 1p/19q co‐deletion in LGG samples from CGGA database, *P* = .0415; with log‐rank test. I‐J, Kaplan‐Meier survival analysis for FABP5 expression in primary I, or recurrent,J LGGs by using CGGA database (I: *P* = .0415, J: *P* = .0415, with log‐rank test). K‐L, Kaplan‐Meier survival analysis for FABP5 expression in grade II K, or grade III L, LGGs by using CGGA database (K: *P* = .2119, L: *P* < .0001, with log‐rank test)

As previously described, FABP5 was a valid indicator for pathological classification and molecular grading of LGGs. To further investigate the prognostic characters of FABP5 in LGGs, Kaplan‐Meier analysis was performed by using our LGG samples as well as open access datasets. Consistent with other previous studies, IDH^WT^ and 1p/19q^Non‐co‐del^ samples showed more severe outcomes compared with samples exhibiting other molecular subtypes (Figure 3A). As shown in Figure 3B, LGG patients with lower expression of FABP5 represented a favourable outcome when compared to those with higher FABP5 expression. Similar results were observed when using data extracted from TCGA and CGGA (Figure 3C,D). Then we combined FABP5 expression with IDH or 1p/19q status as predictors for survival in LGG patients. The results demonstrated that elevated FABP5 combined with either IDH wild‐type or 1p/19q non co‐deletion indicated poor prognosis in LGG patients (Figure 3E,F), which is consistent with the results from CGGA dataset (Figure 3G,H). Furthermore, increased FABP5 was closely correlated with severe prognosis in either primary or recurrence gliomas, particularly among LGGs (Figure S3A,B & Figure 3I,J). Lastly, down‐regulated FABP5 indicated prolonged survival in WHO grade III gliomas and GBM; however, there was no significant correlation between FABP5 and prognosis in WHO grade II gliomas (Figure 3K,L and Figure S3C). Taken together, these findings demonstrated that FABP5 overexpression revealed poor prognosis in LGGs, particularly in malignant LGGs exhibiting high pathological grade or severe subtype based on IDH and 1p/19q status, indicating that FABP5 could serve as a prognostic biomarker and supporting indicator for malignancies in LGGs.

### 3.3 FABP5 was functionally required for multiple malignant behaviours in LGGs

**FIGURE 4:**
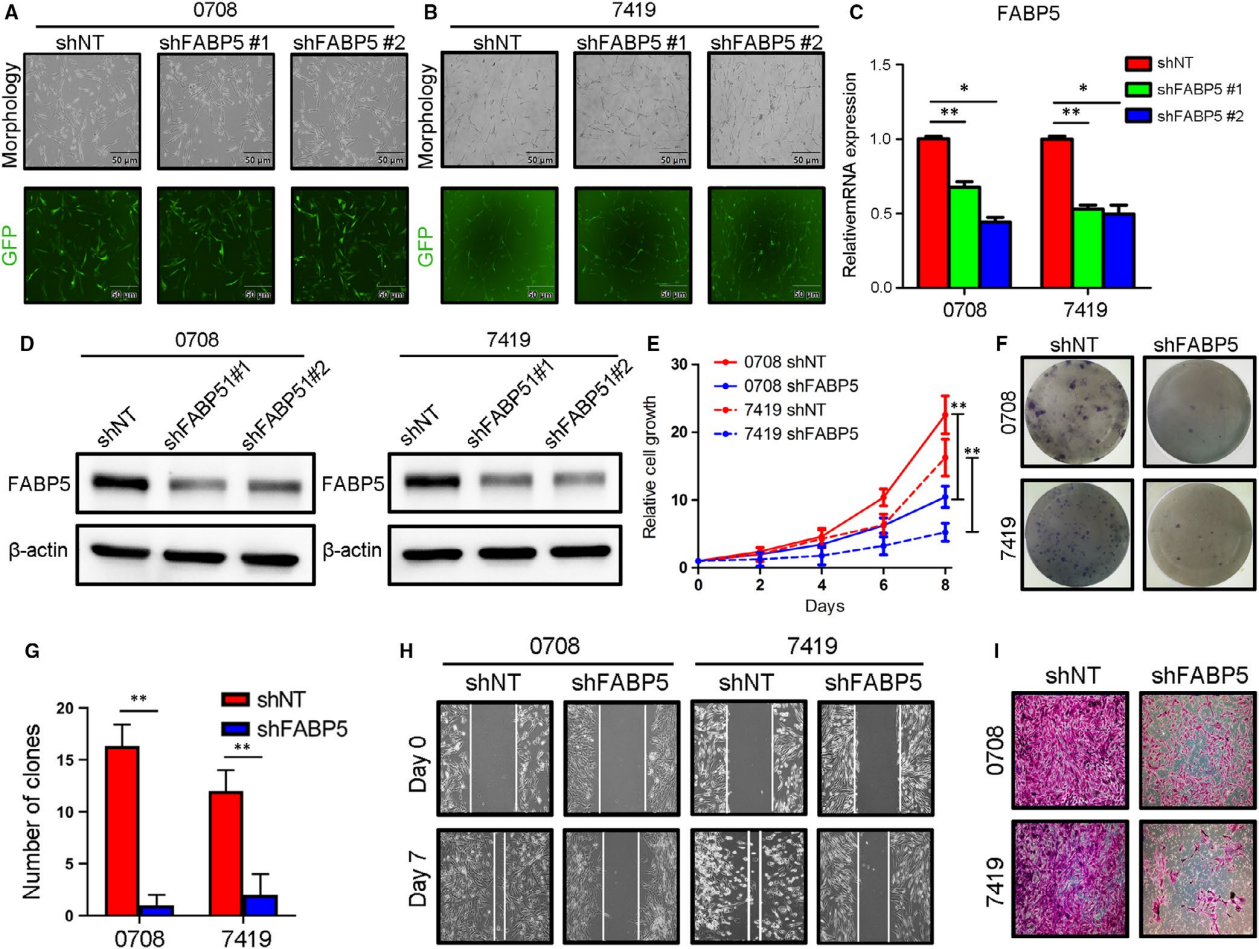
FABP5 was functionally required for multiple malignant behaviours in LGGs. A‐B, Representative fluoresces images for FABP5 knock‐down lentivirus infection in 0708 A, and 7419 B, LGG cells (Upper panel: morphology under microscope, Lower panel: fluoresce images). C, qRT‐PCR analysis of FABP5 in 0708 and 7419 cells transduced with shRNA against FABP5 (shFABP5 #1 and shFABP5 #2) or non‐targeting control (shNT) (*P < .05, **P < .01, n = 3, with one‐way ANOVA followed by Dunnett's post‐test). D, Western blot analysis of FABP5 in 0708 and 7419 cells transduced with shRNA against FABP5 (shFABP5 #1 and shFABP5 #2) or non‐targeting control (shNT). β‐actin served as an internal control (Left panel: 0708 cells, Right panel: 7419 cells). E, In vitro cell growth assay for FABP5 knock‐down (shFABP5) in 0708 and 7419 cells (**P < .01, n = 6, with one‐way ANOVA). F‐G, The colony formation images F, and representative analysis G, of 0708 and 7410 cells pre‐treated with shNT or shFABP5 lentivirus (**P < .01, n = 3, with t test). H, The migration ability of 0708 and 7419 cells pre‐treated with shNT or shFABP5 lentivirus in Day0 and Day3, respectively (Left panel: 0708 cells, Right panel: 7419 cells). I, Invasion ability of 0708 and 7419 cells pre‐treated with shNT or shFABP5 lentivirus (Upper panel: 0708 cells, Lower panel: 7419 cells)

To investigate the function of FABP5 in malignancies of LGGs, an exogenous silencing of FABP5 was performed in early passages of LGGs primary cultures 0708 and 7419 cells via lentivirus infection. The efficiency of transfection was roughly evaluated by fluoresces (Figure 4A,B). qRT‐PCR and western blotting analysis confirmed that FABP5 expression was markedly decreased in FABP5 knock‐down 0708 cells and 7419 cells (Figure 4C,D and Figure S1C). Furthermore, in vitro cell growth assay indicated that the proliferation of 0708 and 7419 cells was obviously reduced by FABP5 silencing (Figure 4E). Moreover, colony formation assays, matrigel invasion assays and wound healing assays were carried out to further study the function of FABP5 on tumour malignant biological behaviour in LGGs. The results indicated that self‐renewal ability, immigration and invasion of LGG cells were remarkably reduced by exogenous suppression of FABP5 (Figure 4F‐I, Figure S1D,E).

**FIGURE 5:**
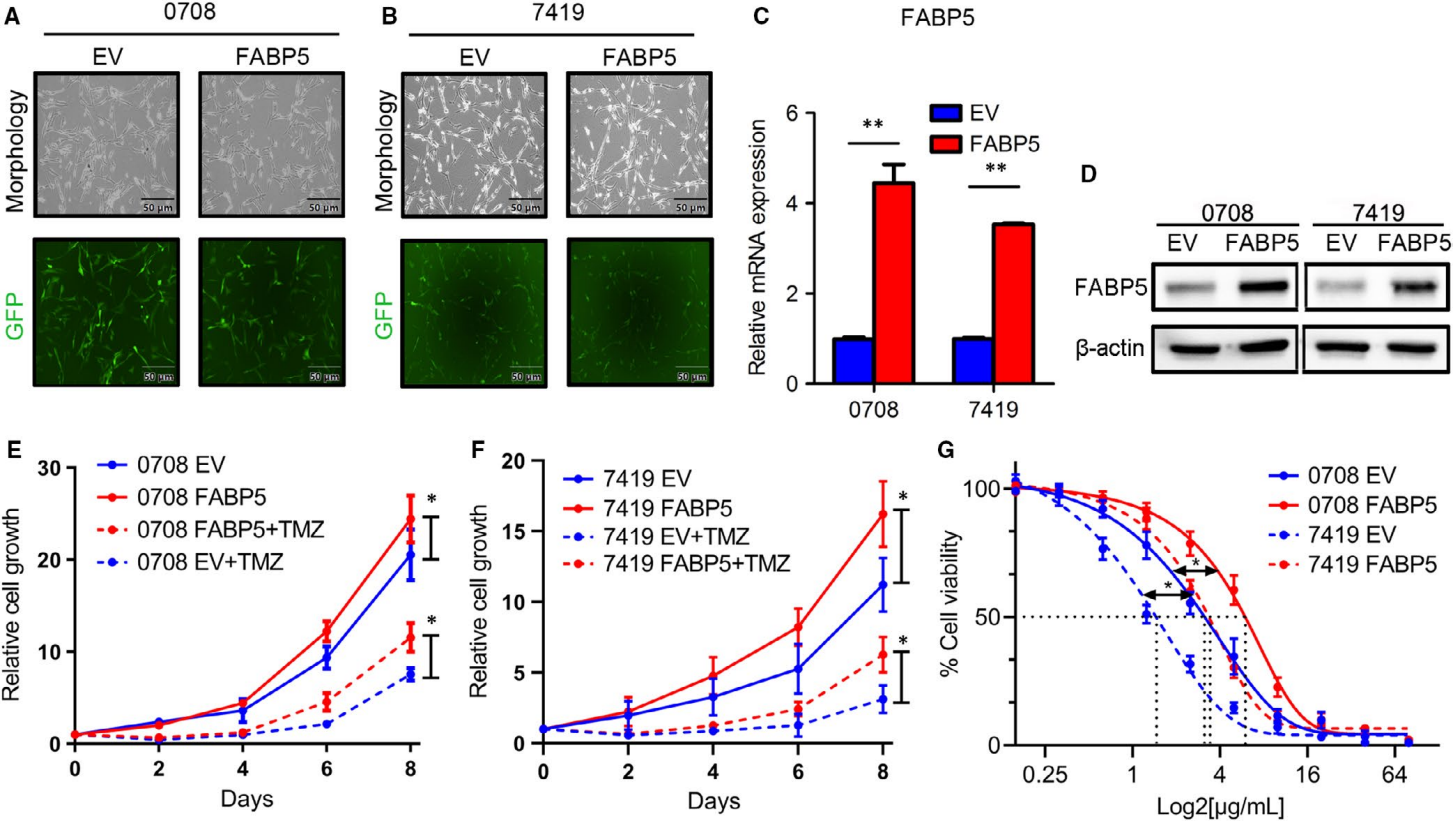
FABP5 overexpression enhanced TMZ resistance in LGG cells. A‐B, Representative fluoresces images for FABP5 overexpression lentivirus infection in 0708 A, and 7419 B, LGG cells (Upper panel: morphology under microscope, Lower panel: fluoresce images). C, qRT‐PCR analysis of FABP5 in 0708 and 7419 cells transduced with FABP5 overexpression (FABP5) or empty vector (EV) lentivirus (**P < .01, n = 3, with t test). D, Western blot analysis of FABP5 in 7419 and 0708 cells transduced with FABP5 overexpression (FABP5) or empty vector (EV) lentivirus. β‐actin served as an internal control. E‐F, In vitro cell growth assay for 0708 E, or 7419 (F) cells pre‐transduced with FABP5 overexpression (FABP5) or empty vector (EV) combined with or without TMZ treatment (*P < .05, n = 6, with one‐way ANOVA). G, In vitro cell toxicity assays for TMZ in 0708 and 7419 cells pre‐transduced with FABP5 overexpression (FABP5) or empty vector (EV) (*P < .05, n = 6, with one‐way ANOVA)

To further confirm the correlation between FABP5 and chemoresistance in LGGs, FABP5 overexpressed 0708 and 7419 cells were constructed before cell viability assays. The efficiency of transfection was roughly evaluated by fluoresces (Figure 5A,B). Results of qRT‐PCR and western blot assays indicated that FABP5 was significantly up‐regulated after lentivirus infection (Figure 5C,D and Figure S1F).

The proliferation assays were then performed to investigate the function of FABP5 overexpression in LGG cells. The result showed that elevated FABP5 significantly enhanced the proliferation and TMZ resistance of 0708 and 7419 cells (Figure 5E,F). Moreover, in vitro cell viability assays indicated that IC50 to TMZ was markedly increased by exogenous FABP5, indicating that overexpression of FABP5 increased the TMZ resistance in LGG cells (Figure 5G). We compare the survival between patients underwent TMZ or not to identify whether FABP5 enhanced the resistance of TMZ. The results showed the high expression of FABP5 with TMZ treatment had a poor prognosis compared to the low expression of FABP5 with TMZ treatment (Figure S2A,B). Altogether, these data demonstrated that FABP5 was functionally required for multiple malignant characters including cell proliferation, colony formation ability, immigration, invasion and chemotherapy resistance in LGGs.

### 3.4 FABP5 induced epithelial‐mesenchymal transition (EMT) in LGGs

**FIGURE 6:**
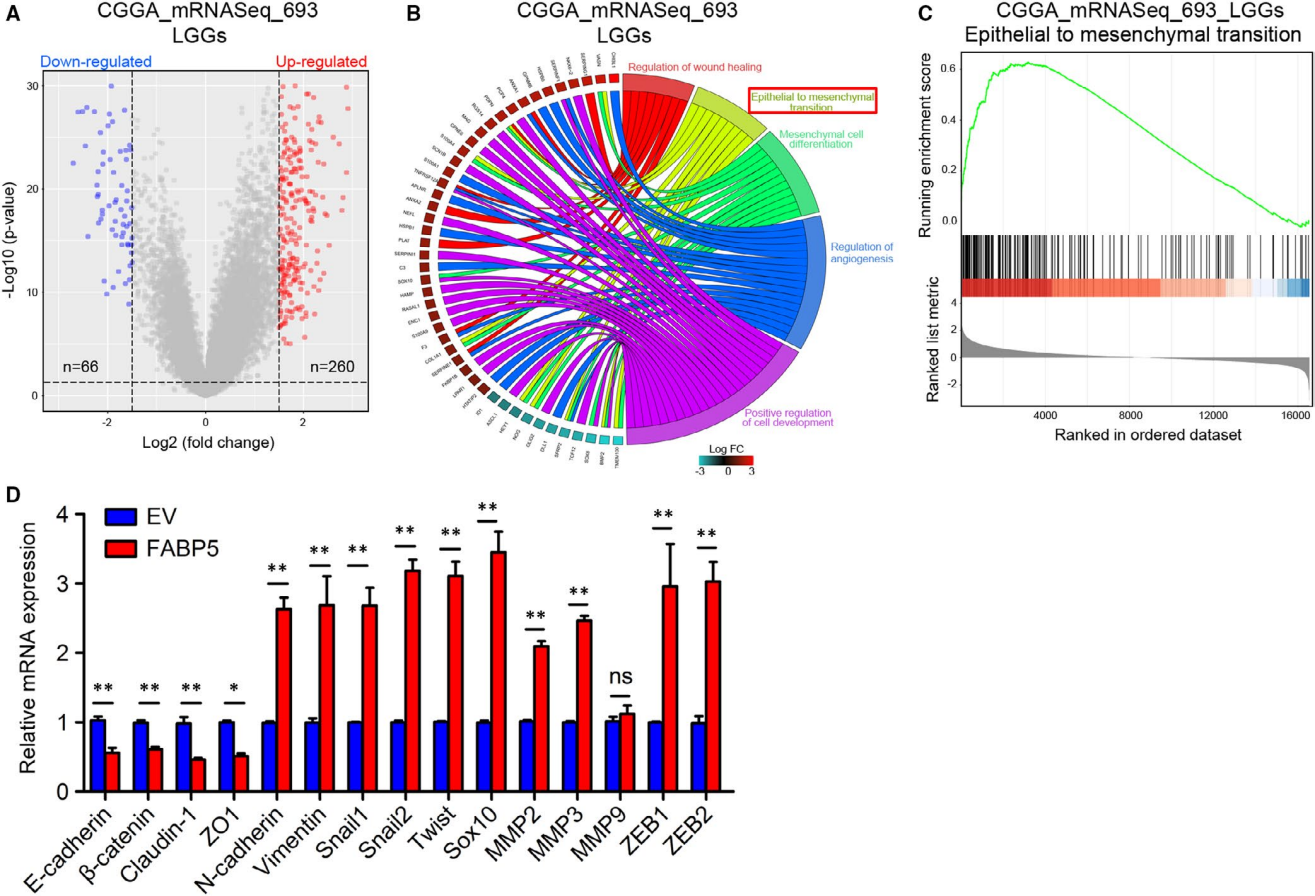
Up‐regulated FABP5 induced EMT in LGGs. A, Volcano plot showed the most significant DEGs in LGGs grouped by FABP5 expression in CGGA database. B, GO annotation analysis based on the FABP5 related DEGs by using CGGA database. C, GSEA results for FABP5 and epithelial‐mesenchymal transition in CGGA database. D, qRT‐PCR assays for expression of the correlated biomarkers of EMT in 0708 cells pre‐transduced with FABP5 overexpression lentivirus (FABP5) and its negative control (EV) (*P < .05, **P < .01, n = 3, with *t* test)

As previously illuminated, FABP5 was enriched in LGGs and promoted malignancies of LGGs, raising the possibility that unknown molecular mechanisms for therapy resistance besides pathological characters need to be clarified. To this end, expression profiles of LGG samples in CGGA and TCGA were grouped into two groups, respectively, depending on FABP5 expression. A subset of DEGs was identified through differential gene expression analysis (Figure 6A and Figure S4A). Then the Database for Annotation, Visualization and Integrated Discovery online tool (DAVID, https://david.ncifcrf.gov/) was used to conduct the GO annotation to investigate FABP5‐related candidate pathways and data were visualized by using clusterprofiler package while GSVA package and GSEA analysis were used as validation. The results indicated that the EMT of GO terms were enriched when analysing either CGGA or TCGA datasets (Figure 6B and Figure S4B). Similarly, GSEA analysis demonstrated that EMT was simultaneously enriched in FABP5^High^ LGGs in both CGGA and TCGA database (Figure 6C and Figure S4C). Furthermore, qRT‐PCR results verified that mesenchymal‐related biomarkers were remarkably elevated by FABP5 overexpression; however, epithelial phenotype was significantly reduced (Figure 6D). In summary, these data revealed that FABP5 induced EMT process, which was possibly responsible for malignant transition of LGGs.

### 3.5 FABP5 enhanced malignancies in LGG via activation of NF‐κB signalling

**FIGURE 7:**
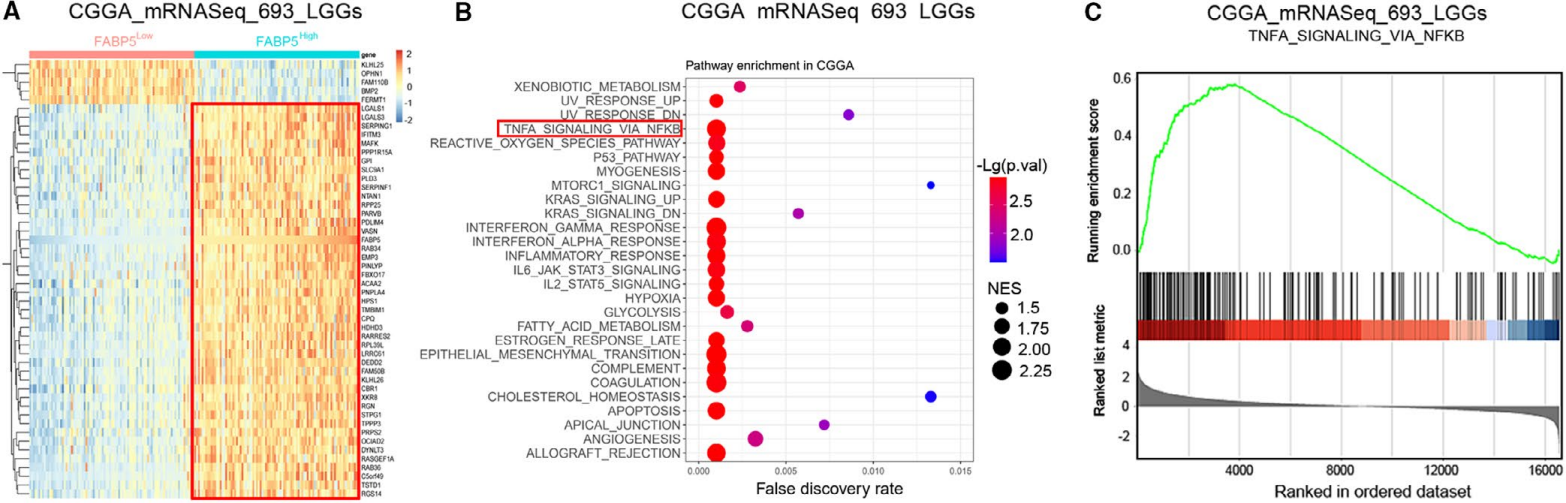
FABP5 expression was correlated with TNFα/NF‐κB signalling in LGGs. A, Hierarchical bi‐clustering analysis with CGGA database indicated that significant gene signatures in LGGs classified by FABP5 expression. B, Bubble plots for GSEA analysis using the transcriptome profiles of LGGs from CGGA database. C, GSEA results for FABP5 and TNFα/NF‐κB signalling in CGGA database

**FIGURE 8:**
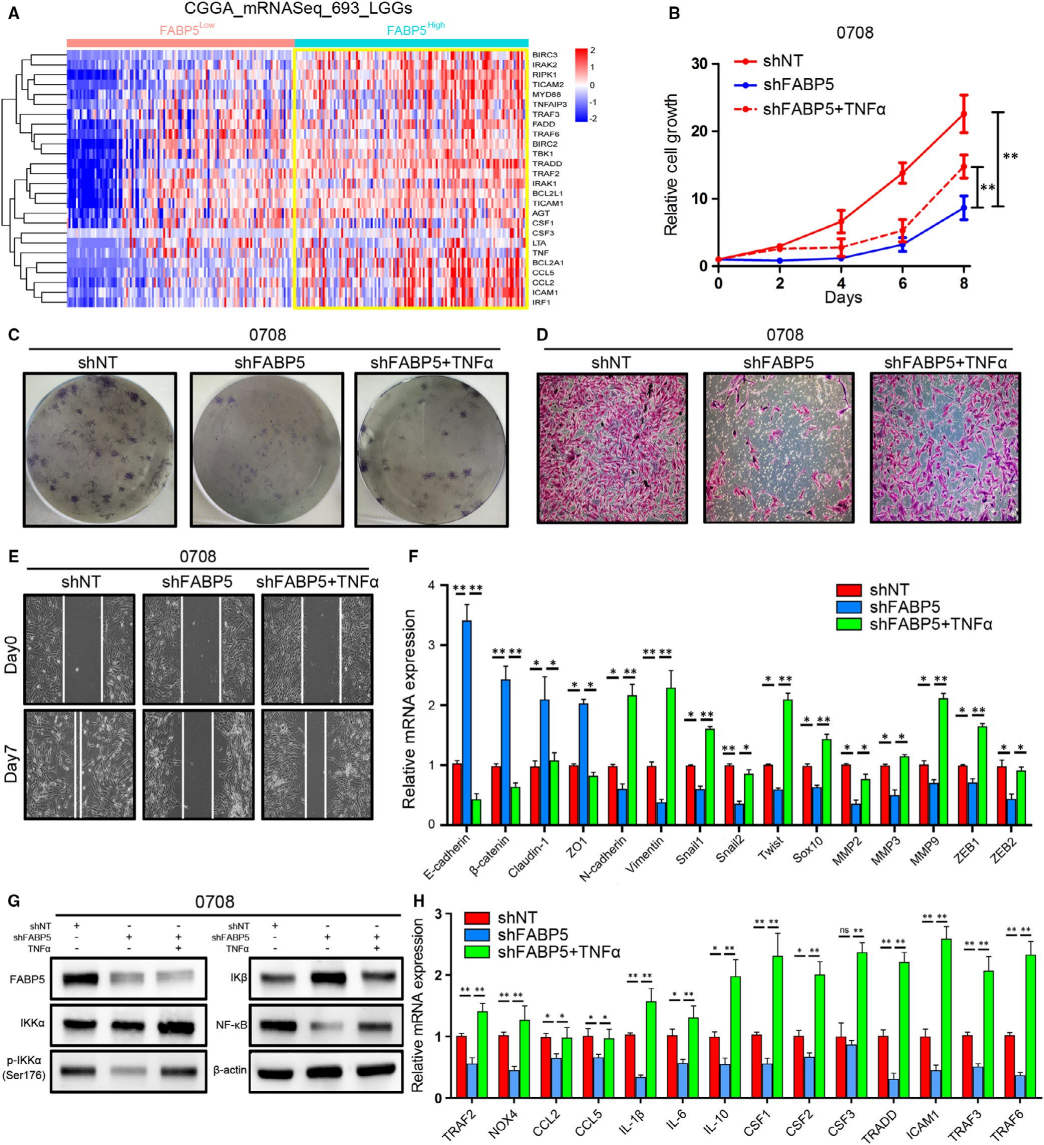
FABP5 was involved in activation of NF‐κB signalling via canonical pathway. A, Hierarchical bi‐clustering analysis for expression level of genes correlated with NF‐κB signalling by using CGGA database. B, In vitro cell growth assay for 0708 cells pre‐transfected with FABP5 knock‐down (shFABP5) or shNT lentivirus followed with or without TNFα treatment (**P < .01, n = 6, with one‐way ANOVA). C‐E, The colony formation assays C, invasion assays D, and migration assays E, for 0708 cells pre‐transfected with FABP5 knock‐down (shFABP5) or shNT lentivirus followed with or without TNFα treatment. F, qRT‐PCR assays for expression of the correlated biomarkers of EMT in 0708 cells pre‐transfected with FABP5 knock‐down (shFABP5) or shNT lentivirus followed with or without TNFα treatment (*P < .05, **P < .01, n = 3, with *t* test). G, Western blot analysis for canonical NF‐κB activators in 0708 cells pre‐transfected with FABP5 knock‐down (shFABP5) or shNT lentivirus followed with or without TNFα treatment. H, qRT‐PCR assays for expression of NF‐κB signalling correlated downstream targets in 0708 cells pre‐transfected with FABP5 knock‐down (shFABP5) or shNT lentivirus followed with or without TNFα treatment (*P < .05, **P < .01, n = 3, with *t* test)

To intensively study the potential downstream molecular pathways of FABP5 in LGGs, GSEA pathway analysis was performed using DEGs from CGGA and TCGA datasets according to the expression of FABP5 (Figure 7A and Figure S5A). Consistently, the TNFα/NF‐κB pathway was simultaneously enriched in FABP5^High^ LGGs in both CGGA and TCGA databases (Figure 7B,C, Figure S5B,C). Moreover, to gain more insights on the potential mechanisms, a bunch of downstream targets correlated to NF‐κB signalling were extracted from CGGA and TCGA databases. Hierarchical bi‐clustering analysis showed that NF‐κB targeting genes were likely to be up‐regulated in FABP5^High^ samples, indicating that NF‐κB signalling could be regulated by FABP5 in LGGs (Figure 8A and Figure S6).

To further validate bioinformatics results and elucidate the effects of FABP5 on regulation of NF‐κB signalling, lentiviral suppression of FABP5 followed with or without TNFα treatment was performed in 0708 LGG cells. In vitro cell growth assays indicated that FABP5 silencing significantly reduced cell proliferation of LGG cells while the cell growth kinetics was partially rescued by the treatment of TNFα (Figure 8B). Similarly, as expected, the ability for colony formation, invasion and migration were also decreased by FABP5 knock‐down and subsequent TNFα treatment exhibited apparent rescue effects against FABP5 silencing (Figure 8C‐E and Figure S1G‐I). In addition, qRT‐PCR analysis showed that FABP5 knock‐down reduced the expression of a wide range of mesenchymal biomarkers; however, TNFα could partially reverse the inhibition effects (Figure 8F). As shown in Figure 8G and Figure S1J, western blot analysis showed that FABP5 knock‐down significantly decreased expression of IKKα, particularly, reduced the phosphorylation level of IKKα thus reduced NF‐κB expression through increased releasing of IKβ. Moreover, FABP5 inhibition notably presented coordinated tendency which reduced downstream targets of NF‐κB pathway, and by contrast, these landmark genes were partially improved on transcriptome and translation levels after TNFα treatment was used (Figure 8H). Altogether, these results suggested that FABP5 induced EMT thus promoted malignancies of LGGs via TNFα‐dependent canonical NF‐κB activation.

## 4 DISCUSSION

Histological prediction of patient survival based on WHO classification has been challenged with the improvement of accurate molecular stratification of gliomas.^20^ Numerous studies demonstrate that IDH mutations induce aberrant DNA histone and methylation thus lead to glioma CpG‐island methylator phenotype (G‐CIMP), which is considered to be correlated with distinct biological and clinical characters in LGGs.^21^, ^22^ Mutant IDH in LGGs acquire a neomorphic enzymatic activity which leads to conversion of α‐ketoglutarate (α‐KG) to D‐2‐hydroxyglutarate (D‐2‐HG), thus suppresses α‐KG‐dependent dioxygenases in LGGs.^23^ Although IDH and the integrity status of 1p/19q is essential for subtype classification and prognosis prediction for LGGs, nevertheless, it is still ambiguous for the underlying mechanism of malignant transition for LGGs.^24^

Recent studies demonstrate that human cells undergo shifting from epithelial subtype to mesenchymal subtype during embryonic development by which those cells modify their adhesion molecules to adopt a migratory or invasive behaviour.^25^ Moreover, mesenchymal subtype transition was also observed in multiple cancers and was proved to be essential for tumour recurrence and therapy resistance.^26^ EMT has been recognized as a crucial promoter of proliferation, migration, metastasis, invasion and therapy resistance in a wide range of malignant cancers.^27^ Accumulating evidence demonstrate that mesenchymal‐like tumour cells dissociated are more likely to invade into the blood vessels and progressed more rapidly compared with the primary tumour.^25^ Similarly, glioma cells will undergo mesenchymal transition which allows the remaining cells to survive from chemotherapy and radiotherapy thus lead to tumour recurrence.^28^ With bioinformatics analysis, we found that FABP5 was closely correlated with EMT. Moreover, exogenous overexpression of FABP5 promoted mesenchymal subtype transition thus induced more rapid cell growth, invasion, immigration as well as TMZ resistance of LGGs, which is consistent with the previous studies. Our study suggested that FABP5‐dependent EMT progress contributed to acquired TMZ chemoresistance in LGGs. We also found that only early passages of LGG primary cells could be used as in vitro cell model; however, these cells underwent cell cycle arrest despite FABP5 overexpression after long‐term in vitro culturing while the in vivo tumorigenesis of LGG cells cannot be further investigated. More efficient research model and experiment methods including patient‐derived xenograft (PDX) model, slice culture and organoid culture should be performed to validate our hypothesis.

NF‐κB is a well‐recognized anti‐apoptotic signalling pathway and aberrant activation of NF‐κB could be observed in multiple types of malignant cancers and contributes to multiple cancer biological behaviours.^29^, ^30^ In cancer cells, NF‐κB is maintained as an inactive form located in the cytoplasm by forming a complex with specific inhibitory proteins including IκB‐α and IκB‐β.^31^ After TNFα binding, TNFRSF1A, which is identified as one of the major TNF receptors, undergoes a conformational modification and allowing them to recruit adapter molecules then leads to activation of NF‐κB signalling.^32^ However, there are various molecular mechanisms which have been proved to be responsible for NF‐κB stimulation in cancer besides the canonical or non‐canonical activation cascade. Therefore, the upstream triggers for NF‐κB pathway still need to be further clarified. FABPs are key intracellular molecules involved in the uptake, transportation and storage of fatty acids and in the mediation of signal transduction and gene transcription.^33^ FABP5 promotes various cancer progression and could be correlated to poor prognosis in a wide range of malignant cancers including cervical cancer, breast cancer, prostate cancer and colorectal cancer, et al^34^, ^35^, ^36^, ^37^ Zhao et al^38^ indicate that specific FABP5 silencing reduces the invasiveness and arrest cell cycle in G0/G1 phase, resulting in a significant increase in apoptosis of gastric cancer cells. Mechanism study reveals that FABP5 silencing reduces intracellular fatty acid (FA) levels then suppressed NF‐κB signalling that leads to reduction of the downstream target genes in breast cancer cells.^39^ Recent studies have indicated that FABP5 plays important roles in the regulation of gene expression associated with cell growth and differentiation since FAs are required as an energy source and for production of cellular signalling molecules and the formation of membrane components during cancer.^39^ However, the functional role for FABP5 in glioma still remains unclear. Our study has indicated for the first time that FABP5 promoted EMT thus enhanced multiple malignant signatures through activation of NF‐κB signalling in LGGs. Mechanistically, phosphorylation level of IKKα and NF‐κB expression was decreased by lentiviral suppression of FABP5, moreover, these inhibition effects could be rescued by TNFα treatment. Although our study demonstrated that FABP5 participate in phosphorylation of IKKα, FABP5 is a fatty acid binding protein and cannot regulate the phosphorylation of NF‐κB, indicating that there might be other intermediate regulators exist as phosphorylation triggers within this process. Mechanism studies including co‐immunoprecipitation and mass spectra analysis need to be done in purpose to clarify the molecular mechanism and specific phosphorylation site of FABP5‐dependent activation of NF‐κB signalling. In conclusion, the present study indicated that FABP5 enhances various malignant signatures in LGGs through activation of NF‐κB signalling and could be used as a prognostic biomarker and therapeutic target for LGGs.

## CONFLICT OF INTEREST

The authors declare no conflict of interest.

## AUTHOR CONTRIBUTIONS

**Yichang Wang:** Data curation (lead); Writing‐original draft (equal); Writing‐review & editing (equal). **Alafate Wahafu:** Formal analysis (equal); Resources (supporting). **Wei Wu:** Formal analysis (equal); Software (equal); Visualization (supporting). **Jianyang Xiang:** Formal analysis (supporting); Investigation (supporting); Validation (supporting). **Longwei Huo:** Conceptualization (supporting); Methodology (supporting); Supervision (supporting). **Xudong Ma:** Data curation (supporting); Formal analysis (supporting); Validation (supporting). **Ning Wang:** Conceptualization (supporting); Methodology (supporting); Project administration (supporting). **Hao Liu:** Project administration (equal); Supervision (equal); Validation (equal). **Xiaobing Bai:** Investigation (supporting); Project administration (supporting); Supervision (supporting); Validation (supporting). **Dongze Xu:** Data curation (supporting); Formal analysis (supporting); Validation (supporting). **Wanfu Xie:** Conceptualization (supporting); Project administration (supporting); Supervision (supporting); Validation (supporting). **Maode Wang:** Conceptualization (equal); Project administration (equal); Supervision (equal). **Jia Wang:** Conceptualization (lead); Data curation (equal); Formal analysis (equal); Funding acquisition (equal); Investigation (supporting); Methodology (equal); Project administration (lead); Resources (lead); Supervision (lead); Validation (equal); Writing‐original draft (lead); Writing‐review & editing (lead).

## Supporting information

Figure S1

Figure S2

Figure S3

Figure S4

Figure S5

Figure S6

Figure S7

Table S1‐S3

Supplementary Material

## Data Availability

The data that support the findings of this study are available from the corresponding author upon reasonable request.
