# Supplementary figures and images for: FABP5 enhances malignancies of lower‐grade gliomas via canonical activation of NF‐κB signaling
Source: J Cell Mol Med. 2021 Apr 9;25(9):4487–500. doi: 10.1111/jcmm.16536 (PMC8093984; doi:10.1111/jcmm.16536)

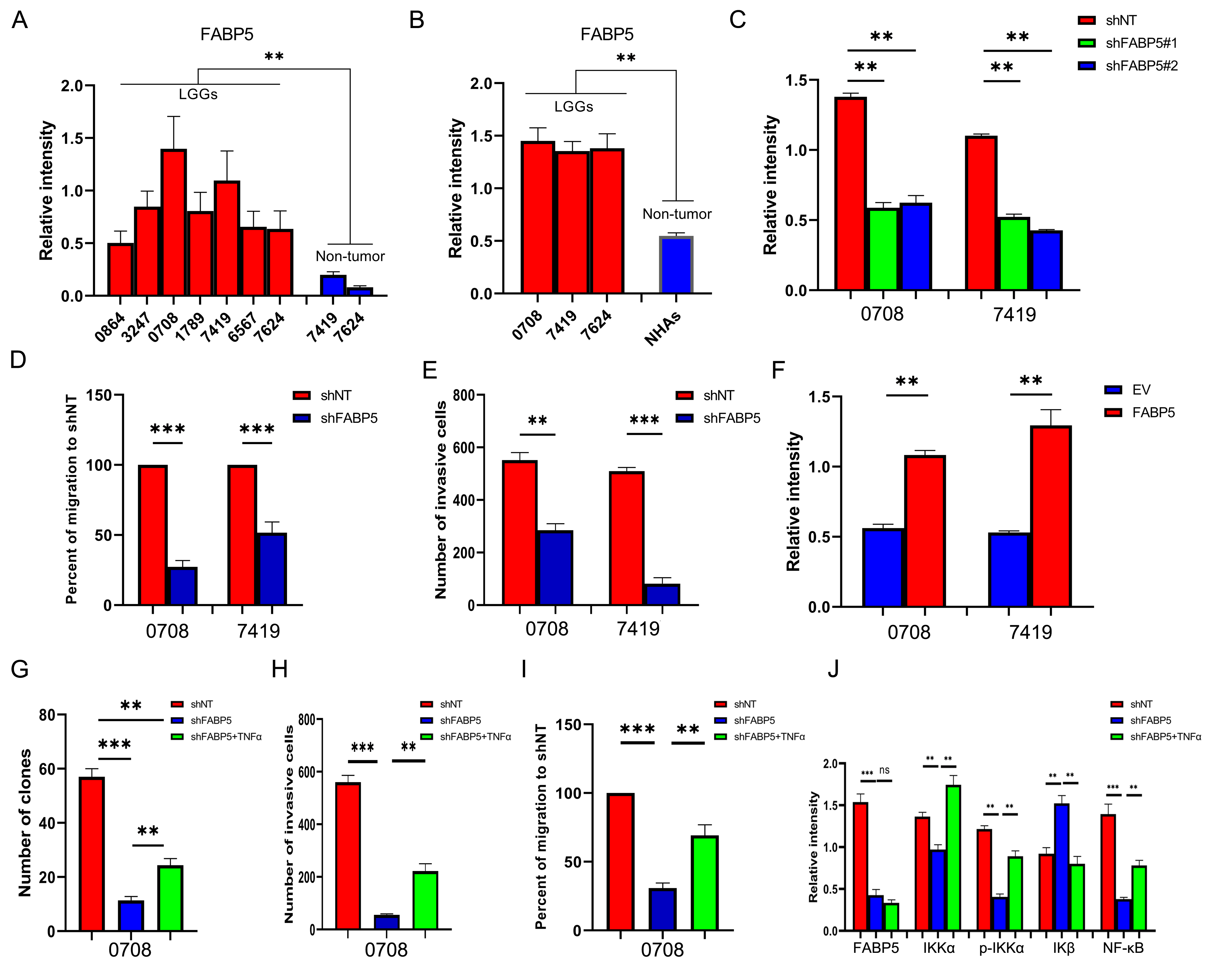

Supplement: Supplementary file 1 — Figure S1 [file JCMM-25-4487-s009.tif]

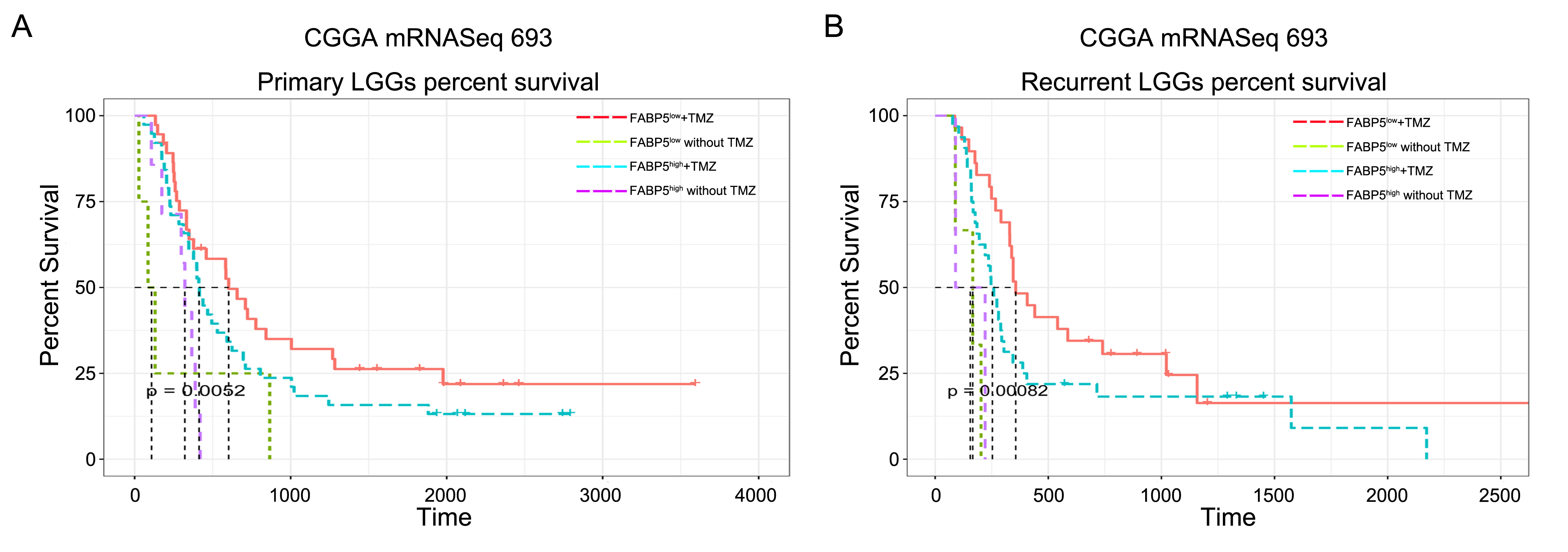

Supplement: Supplementary file 2 — Figure S2 [file JCMM-25-4487-s002.tif]

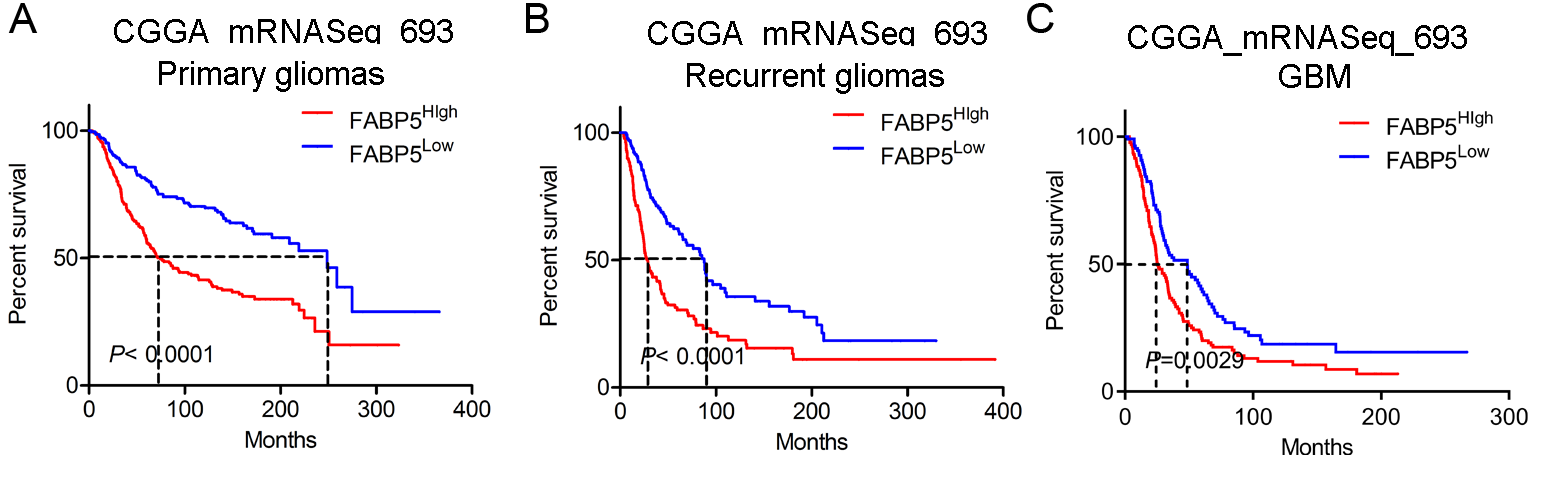

Supplement: Supplementary file 3 — Figure S3 [file JCMM-25-4487-s007.tif]

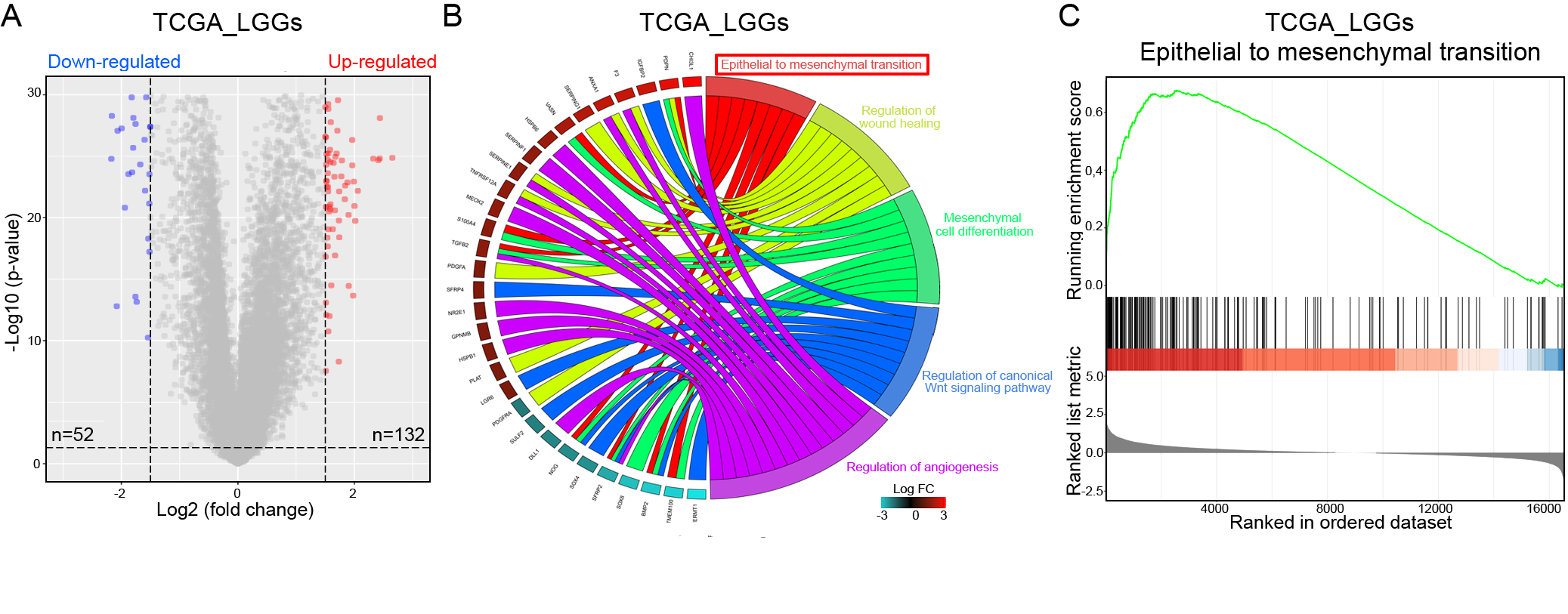

Supplement: Supplementary file 4 — Figure S4 [file JCMM-25-4487-s005.tif]

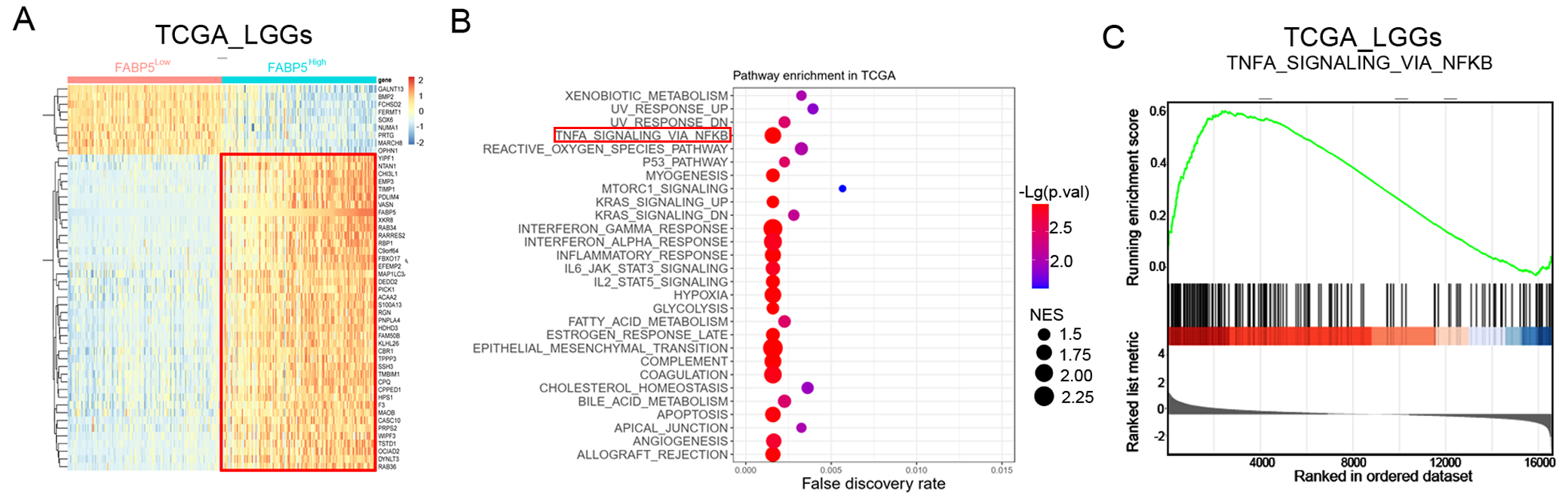

Supplement: Supplementary file 5 — Figure S5 [file JCMM-25-4487-s001.tif]

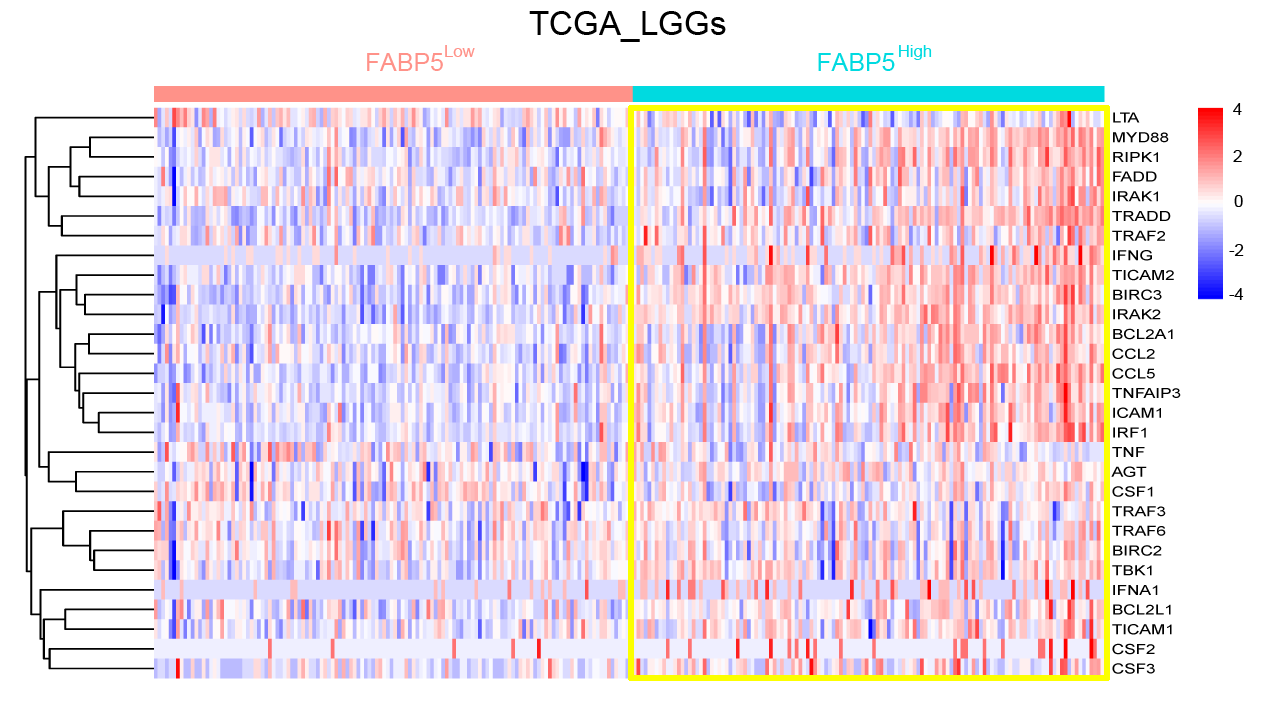

Supplement: Supplementary file 6 — Figure S6 [file JCMM-25-4487-s008.tif]

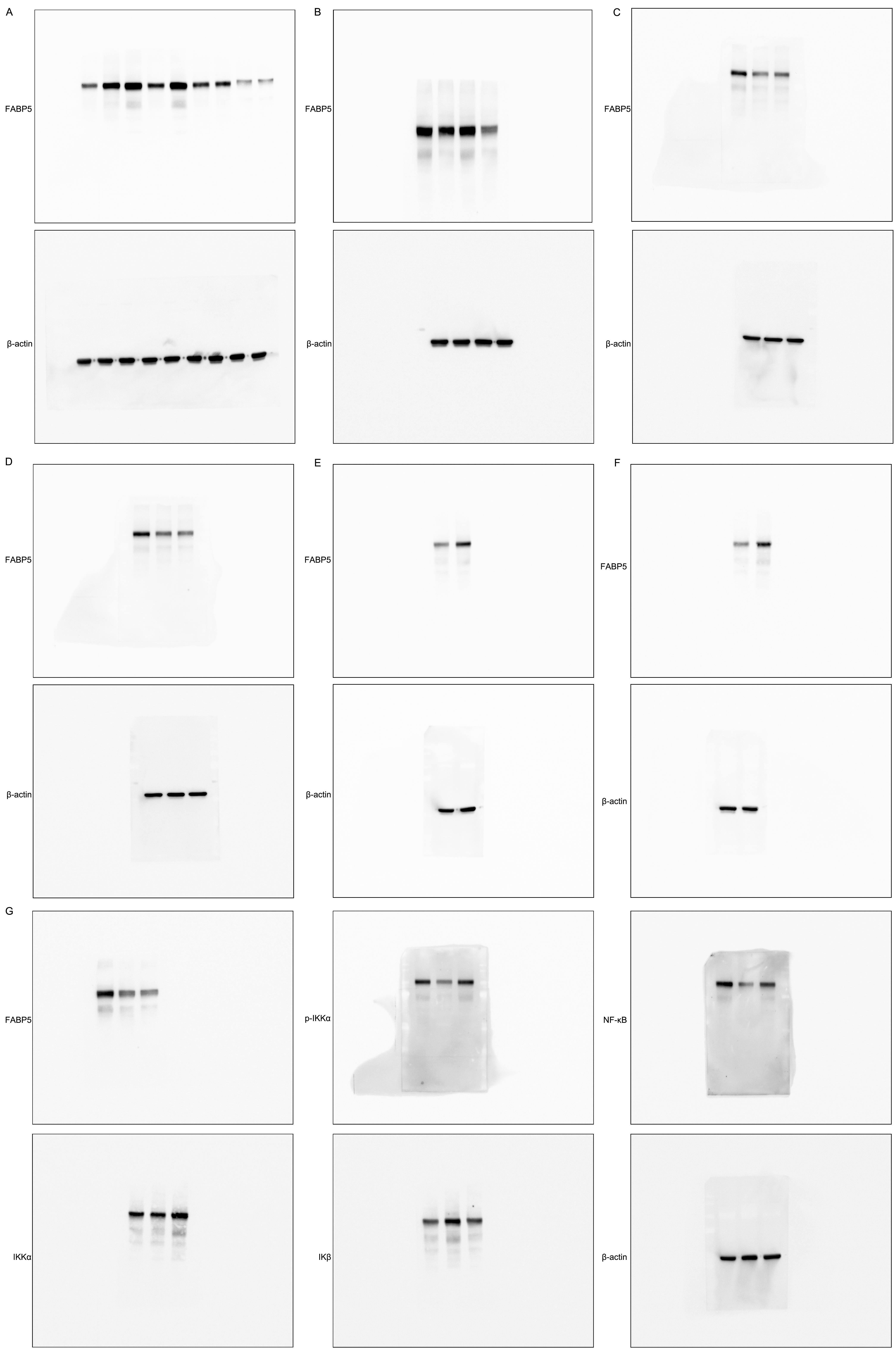

Supplement: Supplementary file 7 — Figure S7 [file JCMM-25-4487-s004.tif]
