# Supplementary material for: FABP5 enhances malignancies of lower‐grade gliomas via canonical activation of NF‐κB signaling
Source: J Cell Mol Med. 2021 Apr 9;25(9):4487–500. doi: 10.1111/jcmm.16536 (PMC8093984; doi:10.1111/jcmm.16536)
Supplement: Supplementary file 8 — Table S1‐S3 [file JCMM-25-4487-s006.docx]

Table. S1 Patient basic information used in this study

| Patient Characters | Patients number |
| --- | --- |
| Total | 63 |
| **Age** | 52.48±11.76 |
| **Gender** |  |
| Male | 28 |
| Female | 35 |
| **WHO Grade** |  |
| II | 29 |
| III | 34 |
| **Tumor Location** |  |
| Frontal lobe | 21 |
| Temporal lobe | 20 |
| Occipital lobe | 11 |
| Parietal lobe | 11 |
|  | |

Table. S2 WHO grade, 1p/19q and IDH status of patient samples used in the study

| Patient sample | 1p/19q status | | IDH status | | WHO grade |
| --- | --- | --- | --- | --- | --- |
| 0864 | Co-deletion | | Mutation | | II |
| 3247 | Non-co-deletion | | Wild type | | III |
| 0708 | Non-co-deletion | | Wild type | | III |
| 1789 | Non-co-deletion | | Mutation | | II |
| 7419 | Non-co-deletion | | Wild type | | III |
| 6567 | Non-co-deletion | | Mutation | | II |
| 7624 | Co-deletion | | Wild type | | III |
|  | |  | |  | |

Table. S3 Sequences of primers used in this study.

| Primer name | Sequence |
| --- | --- |
| FABP5-foward | 5′-AGTTCAGCAGCTGGAAGGAAG-3’ |
| FABP5-reverse | 5′-CGCCCATTTTTCGCAAAGCTA-3’ |
| E-cadherin-forward | 5′-CGAGAGCTACACGTTCACGG-3’ |
| E-cadherin-reverse | 5′-GGGTGTCGAGGGAAAAATAGG-3’ |
| Claudin-1-forward | 5′-CTGGGAGGTGCCCTACTTTG-3’ |
| Claudin-1-reverse | 5′-ACACGTAGTCTTTCCCGCTG-3’ |
| ZO1-forward | 5′-CAACATACAGTGACGCTTCACA-3’ |
| ZO1-reverse | 5′-CACTATTGACGTTTCCCCACTC-3’ |
| N-cadherin-forward | 5’-TCAGGCGTCTGTAGAGGCTT-3’ |
| N-cadherin-reverse | 5’-CATTTCACGCATCTGGCGTTC-3’ |
| Vimentin-forward | 5’-AGTCCACTGAGTACCGGAGAC-3’ |
| Vimentin-reverse | 5’-CATTTCACGCATCTGGCGTTC-3’ |
| Snail1-forward | 5’-TCGGAAGCCTAACTACAGCGA-3’ |
| Snail1-reverse | 5’-AGATGAGCATTGGCAGCGAG-3’ |
| Snail2-forward | 5’-CGAACTGGACACACATACAGTG-3’ |
| Snail2-reverse | 5’-CTGAGGATCTCTGGTTGTGGT-3’ |
| Twist-forward | 5’-GTCCGCAGTCTTACGAGGAG-3’ |
| Twist-reverse | 5’-GCTTGAGGGTCTGAATCTTGCT-3’ |
| Sox10-forward | 5’-CCTCACAGATCGCCTACACC-3’ |
| Sox10-reverse | 5’-CATATAGGAGAAGGCCGAGTAGA-3’ |
| MMP2-forward | 5’-TACAGGATCATTGGCTACACACC-3’ |
| MMP2-reverse | 5’-GGTCACATCGCTCCAGACT-3’ |
| MMP3-forward | 5’-CTGGACTCCGACACTCTGGA-3’ |
| MMP3-reverse | 5’-CAGGAAAGGTTCTGAAGTGACC-3’ |
| MMP9-forward | 5’-AGACCTGGGCAGATTCCAAAC-3’ |
| MMP9-reverse | 5’-CGGCAAGTCTTCCGAGTAGT-3’ |
| ZEB1-forward | 5’-TTACACCTTTGCATACAGAACCC-3’ |
| ZEB1-reverse | 5’-TTTACGATTACACCCAGACTGC-3’ |
| ZEB2-forward | 5’-CCTCTGTAGATGGTCCAGTGAAG-3’ |
| ZEB2-reverse | 5’-GTCACTGCGCTGAAGGTACT-3’ |
| TRAF2-forward | 5’-GCTCATGCTGACCGAATGTC-3’ |
| TRAF2-reverse | 5’-GCCGTCACAAGTTAAGGGGAA-3’ |
| NOX4-forward | 5’-CAGATGTTGGGGCTAGGATTG-3’ |
| NOX4-reverse | 5’-GAGTGTTCGGCACATGGGTA-3’ |
| CCL2-forward | 5’-CAGCCAGATGCAATCAATGCC-3’ |
| CCL2-reverse | 5’-TGGAATCCTGAACCCACTTCT-3’ |
| IL-1β-forward | 5’-ATGATGGCTTATTACAGTGGCAA-3’ |
| IL-1β-reverse | 5’-GTCGGAGATTCGTAGCTGGA-3’ |
| IL-6-forward | 5’-ACTCACCTCTTCAGAACGAATTG-3’ |
| IL-6-reverse | 5’-CCATCTTTGGAAGGTTCAGGTTG-3’ |
| IL-10-forward | 5’-TCAAGGCGCATGTGAACTCC-3’ |
| IL-10-reverse | 5’-GATGTCAAACTCACTCATGGCT-3’ |
| CSF1-forward | 5’-AGACCTCGTGCCAAATTACATT-3’ |
| CSF1-reverse | 5’-AGGTGTCTCATAGAAAGTTCGGA-3’ |
| CSF2-forward | 5’-TCCTGAACCTGAGTAGAGACAC-3’ |
| CSF2-reverse | 5’-TGCTGCTTGTAGTGGCTGG-3’ |
| CSF3-forward | 5’-GCTGCTTGAGCCAACTCCATA-3’ |
| CSF3-reverse | 5’-GAACGCGGTACGACACCTC-3’ |
| TRADD-forward | 5’-GCTGTTTGAGTTGCATCCTAGC-3’ |
| TRADD-reverse | 5’-CCGCACTTCAGATTTCGCA-3’ |
| ICAM1-forward | 5’-TTGGGCATAGAGACCCCGTT-3’ |
| ICAM1-reverse | 5’-GCACATTGCTCAGTTCATACACC-3’ |
| TRAF3-forward | 5’-GCTGTCCTGACAGAAGAGAACT-3’ |
| TRAF3-reverse | 5’-TTTAGCGGCGGGTTAGTCTG-3’ |
| TRAF6-forward | 5’-TTTGCTCTTATGGATTGTCCCC-3’ |
| TRAF6-reverse | 5’-CATTGATGCAGCACAGTTGTC-3’ |
| GAPDH-forward | 5’-ACCCAGAAGACTGTGGATGG-3’ |
| GAPDH-reverse | 5’-TTCAGC TCAGGGATGACCTT-3’ |
