# Supplementary material for: FABP5 enhances malignancies of lower‐grade gliomas via canonical activation of NF‐κB signaling
Source: J Cell Mol Med. 2021 Apr 9;25(9):4487–500. doi: 10.1111/jcmm.16536 (PMC8093984; doi:10.1111/jcmm.16536)
Supplement: Supplementary file 9 — Supplementary Material [file JCMM-25-4487-s003.docx]

Figure S1 Quantitative analysis for Western blot, colony formation assay, wound healing assay and matrigel invasion assays

(A) Western blot analysis of FABP5 in LGG samples (0864, 3247, 0708, 1789, 7419, 6567 and 7624) compared with non-tumour samples deriving from the matched para-cancerous tissues (7419 and 7624) by ImageJ software. (B) Western blot analysis of FABP5 in early passages of patient tumour-derived LGGs primary cultures (0708, 7419 and 7624) compared to normal human astrocytes by ImageJ software. (C) Western blotting analysis of FABP5 expression in FABP5 knock-down 0708 cells and 7419 cells by ImageJ software. (D) The wound areas of 0708 and 7419 cells pre-treated with shNT or shFABP5 lentivirus were measured with ImageJ software and results were plotted on the graphs. The data are represented as the mean of percentage of the wound closure relative to shNT ±SD (n ≥ 3). (E) The invasion analysis of 0708 and 7419 cells pre-treated with shNT or shFABP5 lentivirus. (F) Western blot analysis of FABP5 in 7419 and 0708 cells transduced with FABP5 overexpression (FABP5) or empty vector (EV) lentivirus by ImageJ software. (G) The colony formation analysis, (H) The invasion analysis and (I) Wound areas of 0708 cells pre-transfected with FABP5 knock-down (shFABP5) or shNT lentivirus followed with or without TNFα treatment. (J) Western blot analysis for expression of NF-κB signalling correlated downstream targets in 0708 cells pre-transfected with FABP5 knock-down (shFABP5) or shNT lentivirus followed with or without TNFα treatment. **P* < 0.05; ***P* < 0.01; ****P* < 0.001.

Figure S2 Enriched FABP5 revealed severe prognosis in LGGs with TMZ treatment

(A-B) Kaplan-Meier survival analysis for FABP5 expression combined with TMZ treatment in primary (A) or recurrent (B) LGGs by using CGGA database (*P*<0.001, with log-rank test).

Figure S3 Survival analysis for glioma samples from CGGA database.

(A-B) Kaplan-Meier survival analysis for FABP5 expression in primary (A) or recurrent (B) gliomas by using CGGA database (*P*<0.0001, with log-rank test). (C) Kaplan-Meier survival analysis for FABP5 expression in GBMs by using CGGA database (K: *P*=0.0029, with log-rank test).

Figure S4 Up-regulated FABP5 induced EMT in LGGs.

(A) Volcano plot showed the most significant DEGs in LGGs grouped by FABP5 expression in TCGA database. (B) GO annotation analysis based on the FABP5 related DEGs by using TCGA database. (C) GSEA results for FABP5 and epithelial-mesenchymal transition in TCGA database.

Figure S5 FABP5 was correlated to TNFα/NF-κB signalling in LGGs using TCGA database.

(A) Hierarchical bi-clustering analysis with TCGA database indicated that significant gene signatures in LGGs classified by FABP5 expression. (B) Bubble plots for GSEA analysis using the transcriptome profiles of LGGs from TCGA database. (C) GSEA results for FABP5 and TNFα/NF-κB signalling in TCGA database.

Figure S6 Expression of NF-κB signalling related genes in LGGs grouped by FABP5 expression by using TCGA database.

Figure S7 The raw unedited bands of all western blot images in the manuscript.

(A) Full uncut gel for Figure 1I. (B) Full uncut gel for Figure 1K.(C) and (D) Full uncut gel for Figure 4D of FABP5 in 0708 and 7419 cells transduced with shRNA against FABP5 (shFABP5 #1 and shFABP5 #2) or non-targeting control (shNT). β-actin served as an internal control. (E) and (F) Full uncut gel for Figure 5D of FABP5 in 7419 and 0708 cells transduced with FABP5 overexpression (FABP5) or empty vector (EV) lentivirus. β-actin served as an internal control. (G) Full uncut gel for Figure 8G.
